# Chest computed tomography with multiplanar reformatted images for diagnosing traumatic bronchial rupture: a case report

**DOI:** 10.1186/cc6109

**Published:** 2007-09-03

**Authors:** Morgan Le Guen, Catherine Beigelman, Belaid Bouhemad, Yang Wenjïe, Frederic Marmion, Jean-Jacques Rouby

**Affiliations:** 1 Department of Anesthesiology and Critical Care Medicine, Surgical Intensive Care Unit Pierre Viars and the Trauma Center, La Pitié-Salpêtrière Hospital, Assistance Publique Hôpitaux de Paris, University Pierre et Marie Curie Paris-6, France; 2 Department of Radiology, Surgical Intensive Care Unit Pierre Viars and the Trauma Center, La Pitié-Salpêtrière Hospital, Assistance Publique Hôpitaux de Paris, University Pierre et Marie Curie Paris-6, France

## Abstract

**Introduction:**

Unnoticed bronchial injury during the early stage of resuscitation of multiple trauma is not rare and increases mortality and morbidity.

**Methods:**

Three-dimensional reconstruction of the airways using a workstation connected to a multidetector chest computed tomography (CT) scanner may change the diagnostic strategy in patients with blunt chest trauma with clinical signs evocative of bronchial rupture.

**Results:**

In this case report of a young motor biker, a complete disruption of the intermediary trunk was first misdiagnosed using standard chest helical CT and bronchoscopy. Postprocessing procedures including three-dimensional extraction of the tracheobronchial tree were determinants for establishing the diagnosis, and emergent surgical repair was successfully performed. Follow-up using CT with three-dimensional reconstructions evidenced a bronchial stenosis located at the site of the rupture.

**Conclusion:**

The present study demonstrates the potential interest of performing three-dimensional reconstructions by extraction of the tracheal–bronchial tree in patients with severe blunt chest trauma suspected of bronchial rupture.

## Introduction

Tracheobronchial injuries, although rarely observed following blunt chest trauma [1-3], are associated with a mortality ranging between 9% and 30% [3-5]. Traumatic injury of the airways is suspected in the presence of subcutaneous cervical emphysema expanding with mechanical ventilation, pneumomediastinum and recurrent pneumothorax due to a persisting air leak [6]. To date, tracheobronchoscopy remains the reference diagnostic tool [1,6-11]. The procedure, however, is accurate only when performed by trained thoracic or trauma surgeons and pneumologists [7,12]. Moreover, the tracheobronchial injury may be very difficult to diagnose even by an experienced practitioner. As a consequence, tracheobronchial injury may go unnoticed during the early stage of resuscitation and can lead to increased mortality [5] and morbidity through recurrent pneumonia, mediastinitis and atelectasis delaying the mechanical ventilation withdrawal [8,13].

Chest computed tomography (CT) is considered the more relevant diagnostic tool in hemodynamically stable patients with blunt chest trauma following the basic and essential chest X-ray scan. CT has a significant therapeutic impact [14]. Multidetector CT provides high spatial resolution images of the whole lung without any anatomical gap. The postprocessing procedure mainly requires a minimum-intensity projection technique for airway imaging. The technique consists of projecting the voxels with the lowest attenuation value in every view through the volume explored, at various angles depending on the airway involved. If tracheobronchial injury is suspected, three-dimensional (3D) extraction of the airways may be useful by focusing the 3D volume-rendering technique on the tracheobronchial tree. This technique is classically used for analyzing stenosis or distortion of the tracheobronchial tree but may also allow the diagnosis of tracheobronchial injury by demonstrating a wall defect and/or an abnormal position of lobar and segmental bronchi [15]. Surprisingly, reports on the use of CT for diagnosing traumatic tracheobronchial rupture are scarce [16-18] and show disappointing results [16].

The present clinical report demonstrates that chest CT with 3D reconstruction of the tracheobronchial tree may be of unique value for the emergency diagnosis of bronchial rupture.

## Case report

**Figure 1:**
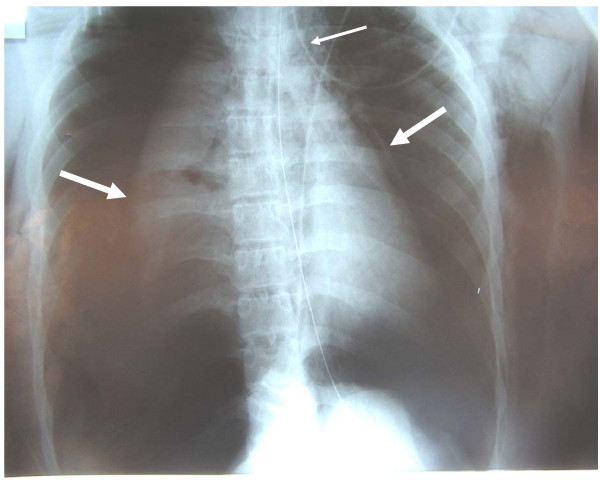
Bedside chest radiography performed immediately after admission. Bilateral pneumothorax (large arrows), pneumomediastinum (thin arrows) and extensive subcutaneous emphysema are visible.

A 19-year-old motor biker was involved in a high-velocity accident against a fixed obstacle. At the scene, the patient was unconscious (Coma Glasgow Scale = 5/15) and severely hypoxemic (oxygen saturation = 80%) with a cervicothoracic emphysema. The patient was intubated, mechanically ventilated and transported to our Level I Trauma Centre. As shown in Figure 1, bedside frontal chest radiography showed bilateral and compressive pneumothorax, pneumomediastinum and extensive subcutaneous emphysema. Arterial oxygenation immediately improved following emergency chest tube placement, and a new chest radiography showed incomplete re-expansion of the right lung with a persistent air leak despite continuous suction.

**Figure 2:**
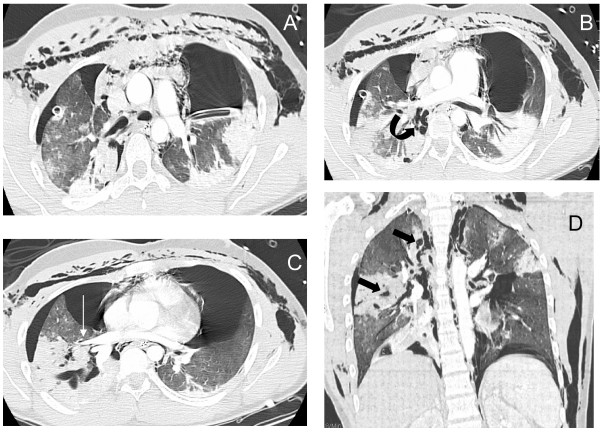
Computed tomography scan following emergency chest tube drainage. Axial 1.25 mm thick sections with a lung window. **(a) **Persistent bilateral pneumothorax, pneumomediastinum and extensive subcutaneous emphysema. **(b) **Multiple lucencies around the right bronchial tree (curved arrow) precluding the correct recognition of the bronchial rupture. **(c) **The Macklin effect around the right lower pulmonary vein (white arrow). **(d) **Coronal view demonstrating multiple areas of alveolar consolidation in the right upper and lower lobes: intraparenchymal lucencies resulting from lung lacerations are visible on the right side (thick arrows).

The patient was then transported to the Department of Radiology for a total body scan (16 slices; Lightspeed GE, General Electric, Milwaukee, WI, United States of America)). The following injuries were diagnosed: brain damage, related to a left parietal contusion with mild subarachnoid hemorrhage (Fisher II); and bilateral pneumothorax with a small hemothorax predominating on the left side, pneumomediastinum, pulmonary interstitial emphysema (Macklin effect [19]), pneumopericardium, subcutaneous emphysema and multiple rib fractures (Figure 2). It has to be pointed out that the right-upper-lobe bronchus was displaced posteriorly without a characteristic CT fallen sign as described by Tack and colleagues [18]. Other concomitant injuries were: myocardial contusion, diagnosed as the presence of sinus tachycardia with an anterior and septal elevation of the ST-segment on EKG (electrocardiogram) and an initial cardiac troponin I value of 10.25 IU (normal value, <0.04 IU); fracture of the first thoracic vertebra without neurological consequence; and right femoral fracture. Orthopedic surgical repair was performed without delay.

Tracheobronchial injury was suspected because the bedside chest radiography performed after orthopedic surgery showed persisting right apical pneumothorax with continuous air leakage and extensive cervicothoracic emphysema. The diagnosis, however, could not be confirmed by bronchoscopy because of the rapid drop in oxygen saturation and the abundant bleeding of the respiratory tract. Consequently, a new CT scan was performed using a technique specifically aimed at visualizing the airways. After contrast material injection, 1.25 mm CT sections at 0.6 mm intervals were acquired and 3D images were obtained following multiplanar reformation.

**Figure 3:**
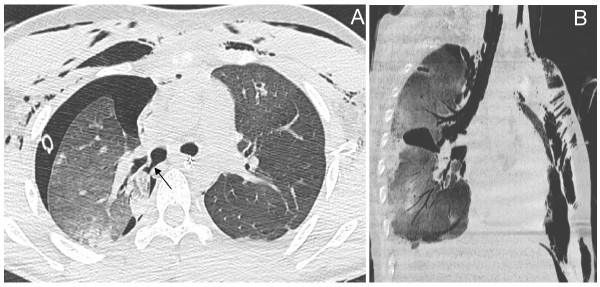
Second thoracic computed tomography scan on day 2 (axial and oblique views). **(a) **The intermediate trunk is disrupted with a visible posterior wall defect below the origin of the right upper lobe bronchus (arrow). Note the persisting right pneumothorax despite adequate chest tube drainage. **(b) **An abnormal lucency raising the possibility of a bronchial disruption is seen on the oblique view.

**Figure 4:**
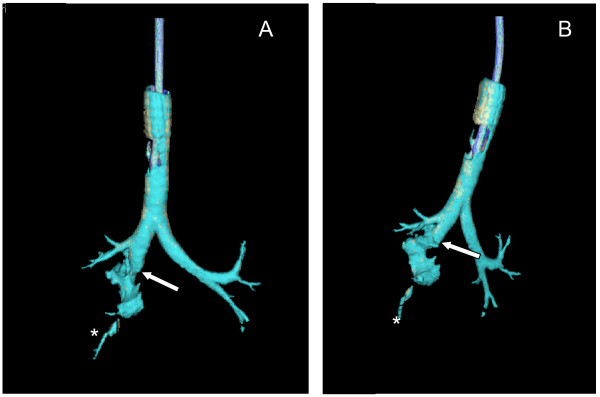
Coronal and oblique views of three-dimensional reconstructions of the tracheobronchial tree. The **(a) **coronal and **(b) **oblique views demonstrate the disruption of the intermediary trunk with an abnormal lucency connected to it (white arrow) and show the partial visualization of segmental branches of the right-lower-lobe bronchus (*).

The axial slice demonstrated a parietal defect of the posterior wall of the intermediate trunk (Figure 3). Multiple oblique reformations of the right lung and 3D reconstruction of the airways brought definitive evidence of a complete right bronchial disruption just below the origin of the upper right bronchus associated with a partial collapse of the right-middle lobe and of the right-lower lobe (Figure 4). A surgical procedure was decided upon, and confirmed a complete disruption of the right bronchus immediately below the origin of the right upper bronchus with an atelectasis of the right middle and inferior lobes. End-to-end anastomosis of the disrupted bronchus was performed through a right thoracotomy and resulted in an immediate re-aeration of the lower lobe, a cessation of the air leak through the right chest tube and a rapid regression of the subcutaneous emphysema, whereas the right-middle lobe remained atelectatic. The decision to perform anastomosis rather than lung resection was based on the patient's young age, the early diagnosis (<24 hours) and the quality of bronchial tissue. Because an anatomical sleeve was present keeping the lower lobe partially aerated, a re-aeration of the middle lobe was expected after re-establishing bronchial continuity.

A bronchoscopy performed on the second postoperative day demonstrated a watertight suture with local inflammation. The postoperative course was complicated by early ventilator-associated pneumonia caused by *Escherichia coli *leading to acute respiratory distress syndrome. Mechanical ventilation with a limited tidal volume, a limited peak airway pressure and a limited positive end expiratory pressure was delivered according to recent recommendations [20-22]. A second ventilator-associated pneumonia caused by *Pseudomonas aeruginosa *delayed withdrawal of the patient from mechanical ventilation, which was successfully achieved on day 18.

**Figure 5:**
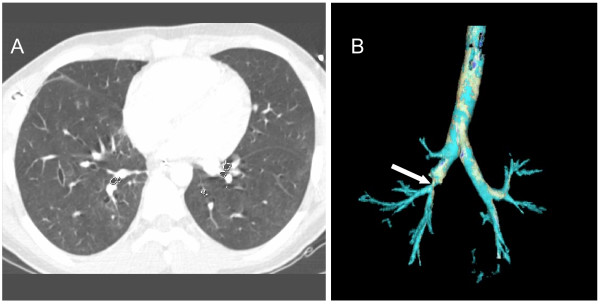
Computed tomography scan performed 2 weeks following surgery. **(a) **Complete recovery of the pulmonary contusion (axial slice at the level of the lower lobes). **(b) **The three-dimensional reconstruction of the tracheobronchial tree, however, demonstrates a bronchial stenosis (white arrow) at the site of surgical repair.

A new CT scan was performed on day 22, before the patient left the intensive care unit. Transversal CT sections demonstrated a normal aeration of the right lung whereas 3D reconstruction of the airways demonstrated a short but tight bronchial stenosis located at the site of the initial rupture (Figure 5). In the absence of new respiratory symptoms, prolonged medical supervision was decided upon and the patient left the intensive care unit on day 28 for a rehabilitation center.

## Results and discussion

Although always symptomatic [23], tracheobronchial injury is a rare entity, not easy to diagnose. The lack of specificity of subcutaneous emphysema, stridor, bronchopleural fistula, pneumomediastinum, hemoptysis, pneumothorax and the occult nature of the injury frequently result in a delayed diagnosis. In addition, associated injuries such as head trauma [24] can mask the diagnosis in the early period following hospital admission, and emergency surgical procedures may also interfere with the diagnostic procedures.

Among all clinical and radiological signs that are frequently observed in tracheobronchial rupture [10,14,25,26], the simultaneous presence of pneumomediastinum and cervical emphysema appears to be the most frequent association [27]. In a retrospective series of 14 patients with confirmed tracheal disruption, the association was observed in each individual patient [27]. In patients with tracheal rupture, the pneumomediastinum results directly from the tracheal air leak. In the present case report, the pneumomediastinum was related to the bronchial rupture into the hilum with a retrograde dissection into the mediastinum. Logically, tracheobronchial rupture should not be associated with pulmonary interstitial emphysema, a radiological sign resulting from alveolar ruptures at the lung periphery [28]. In the present clinical report, a Macklin effect was observed on the initial CT scan, suggesting a peripheral lung barotrauma rather than a bronchial disruption. This finding is in accordance with a previous study that reported the presence of a Macklin effect in a patient with tracheobronchial injury [19], and suggests that alveolar barotrauma and tracheobronchial rupture might be associated in patients with severe blunt chest trauma.

Most trauma centers agree that the diagnosis of tracheobronchial rupture should be confirmed before undertaking surgical repair. Ideally, bronchoscopy preceded by rigid bronchoscopy for clearing blood and secretions from the aiways remains the reference diagnostic tool in patients with blunt chest trauma [10,29-31]. Indeed, airway injuries are mainly located on the initial part of the respiratory track: 19% of ruptures are purely tracheal and 76% are exclusively bronchial, either on the right main stem bronchus (47%) or on the left main stem bronchus (32%) [5]. Bronchoscopy, however, and even more rigid bronchoscopy, is a procedure that requires specific skills, and therefore is not always and easily available under emergency conditions. Endotracheal intubation often precludes the use of rigid bronchoscopy, limiting the procedure and as a consequence limiting bronchoscopy. In the present clinical report, rigid bronchoscopy was not available at admission and the patient was intubated. Although bronchoscopy was performed by an experienced physician, the technical conditions of the procedure were precarious, characterized by abundant bleeding of the respiratory tract and a rapid drop of arterial oxygen saturation, all factors that precluded diagnostic confirmation.

A second lung CT scan was then performed with thinner sections to optimize under specific technical conditions the 3D extraction of the tracheobronchial tree reconstruction (Figure 3). To our knowledge, the present clinical case reports for the first time a right bronchial rupture that could be easily diagnosed using CT 3D reconstruction. In the immediate postinjury period, between 30% and 68% of tracheobronchial ruptures are overlooked by conventional radiographies [32,33]. A few studies have suggested that conventional axial two-dimensional CT is superior to conventional radiographs for diagnosing tracheobronchial rupture [27,34-36]. Two-dimensional CT may evidence pneumomediastinum unsuspected on conventional radiographies, is the reference radiological tool for diagnosing the Macklin effect, and has, theoretically, the ability to identify the site of the tracheobronchial tear [18,27,35].

In a retrospective series of 14 patients with tracheal rupture, the tracheal wall injury was directly visualized on CT as a wall defect or discontinuity in 57% of patients and was indirectly suspected as a tracheal wall deformity in 14% of patients [27]. In fact, reading of axial CT sections by the radiologist requires extensive mental integration and remains challenging even for the experienced practitioner, especially when multiple abnormal lucencies are present. As much as 25% of tracheal ruptures remain undiagnosed using conventional axial CT sections. As previously reported [16], it was impossible for the radiologist to definitively assert the diagnosis of right bronchial rupture on the first CT scan performed in our patient, despite the volumetric acquisition with thin slices on the lung window and multiple reformats. Finally, the diagnosis was made thanks to 3D reconstruction.

In patients with blunt chest trauma and subcutaneous emphysema, with pneumomediastinum, with interstitial pulmonary edema, with 'fallen lung sign' [18,35] and/or with persistent pneumothorax despite adequate drainage [16], we propose the following diagnostic algorithm. A thin-slice CT scan of the chest should be the initial screening tool. If the CT findings are 'evocative' on the axial images, the images should then undergo reformatting and volume subtraction techniques to better define the airway in three dimensions and to rule out artifacts of imaging presenting as 'abnormal lucencies'. If the findings on the reformatted images are still suspicious, or even 'obvious', then the patient should undergo the gold standard test of bronchoscopy. It may be difficult to perform this test in certain patients with airway compromise, but every effort should be made to do so before the patient is subjected to a thoracotomy purely based on the findings of a CT scan reconstruction. One should keep in mind that motion artifacts from the lung and the heart may interfere with the interpretation of the images.

In addition to the diagnosis of upper airway injury, helical CT with 3D reconstruction allows the diagnosis of further tracheobronchial stenosis even with low-dose CT [15,37]. In the present clinical report, a bronchial stenosis at the site of surgical repair was diagnosed 3 weeks after surgery (Figure 5). Again, the single simple examination of axial CT sections overlooked the diagnosis.

## Conclusion

The present study demonstrates the interest of performing 3D reconstructions in patients with severe blunt chest trauma and with clinical symptoms evocative of bronchial rupture undergoing a multislice CT scan. Such a 3D reconstruction may help the clinician to decide to perform a bronchoscopy, which remains the reference diagnostic technique but appears more invasive and risky for the patient. Until well designed prospective studies comparing CT scans and bronchoscopy results are performed, 3D reconstruction should be considered a suitable 'screening' test in a trauma patient suspected of bronchial rupture.

## Key messages

• Care of multiple trauma patients with blunt chest trauma is complex because it increases the risk of unnoticed lesions.

• Development of new software with a helical chest computer may be of serious help in assessment of the tracheobronchial tree. A trained radiologist's interpretation is important due to possible artifacts.

## Abbreviations

CT = computed tomography; 3D = three-dimensional.

## Competing interests

The authors declare that they have no competing interests.

## Authors' contributions

MLG suggested, drafted and promoted this case report with FM's help in analyzing the literature. CB and YW took an active part in the diagnosis, and brought knowledge of choosing images and accurate corrections of the whole radiologic comments. BB and J-JR revised the manuscript for important intellectual content. All authors read and approved the final manuscript.
